# Left Bundle Branch Area Pacing in a Giant Atrium With Atrial Standstill: A Case Report and Literature Review

**DOI:** 10.3389/fcvm.2022.836964

**Published:** 2022-03-29

**Authors:** Jing Zheng, Qingye Yang, Jiasheng Zheng, Qiang Chen, Qizhi Jin

**Affiliations:** Department of Cardiology, The Quzhou Affiliated Hospital of Wenzhou Medical University, Quzhou People’s Hospital, Quzhou, China

**Keywords:** left bundle branch area pacing, physiological pacing, atrial standstill, giant atrium, permanent pacemaker

## Abstract

Atrial standstill (AS) is a rare condition defined by the lack of atrial electrical and mechanical activities. It is usually clinically manifested as symptomatic bradycardia, which requires permanent pacemaker (PPM) implantation. Traditional right ventricular apical pacing causes electrical and mechanical dyssynchrony resulting in left ventricular dysfunction, heart failure, and arrhythmias. As a novel physiological pacing strategy, left bundle branch area pacing (LBBaP) has demonstrated effectiveness and safety in recent years, but its application in exceptional conditions is rarely reported. We report the case of a 47-year-old female, who was diagnosed with AS complicated with a giant atrium, and successfully received a single-chamber PPM with LBBaP.

## Introduction

Atrial standstill (AS) is a rare type of arrhythmia characterized by the loss of electrical and mechanical activities of the atrium (1, 2). Electrocardiogram (ECG) typically shows no visible P waves or atrial fibrillatory waves and a borderline or ventricular escape rhythm. It is clinically characterized by symptomatic bradycardia that requires permanent pacemaker (PPM) therapy. Since AS is always combined with atrial enlargement and tricuspid regurgitation, ventricular lead implantation is a challenge. A single-chamber PPM with its ventricular active lead positioned in the right ventricular apex was traditionally performed in a few previous cases (3, 4). As a physiological pacing strategy, left bundle branch area pacing (LBBaP) was recently proposed. It activates the normal cardiac conduction, thereby providing synchronized contraction of the ventricles (5). However, AS with a giant atrium is a challenge to the placement of the ventricular lead, especially for LBBaP, as similar cases are rarely reported.

## Case Report

**FIGURE 1:**
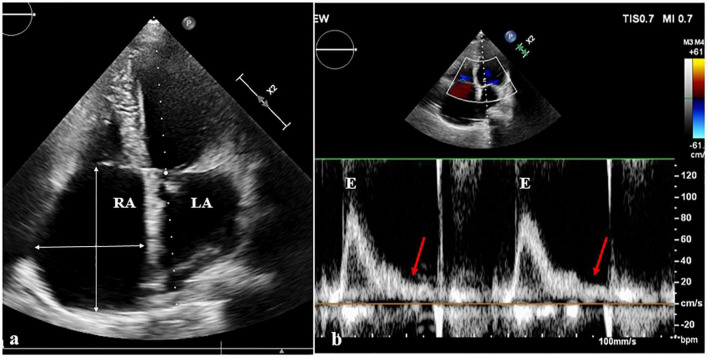
Echocardiography images. **(a)** On the echocardiography, both atrium were observably enlarged, the right atrium (RA) size was 8.9 cm × 5.6 cm, and the left atrium (LA) size was 7.2 cm × 5.6 cm. **(b)** In the mitral inflow pulse by Doppler recording, only E wave was observed in the early diastolic period, but no A wave was observed in the late diastolic period.

**FIGURE 2:**
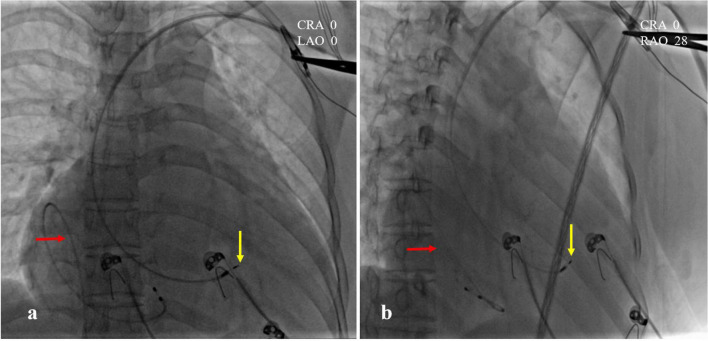
Intraoperative X-ray fluoroscopy images. **(a)** CRA 0, LAO 0; **(b)** CRA 0, RAO 28. The temporary pacemaker lead was coiled in the right atrial lumen (red arrow). The position of left bundle branch area pacing (LBBaP) was at the distal end of the ventricle, 1.5 cm approximately beyond HIS (yellow arrow).

**FIGURE 3:**
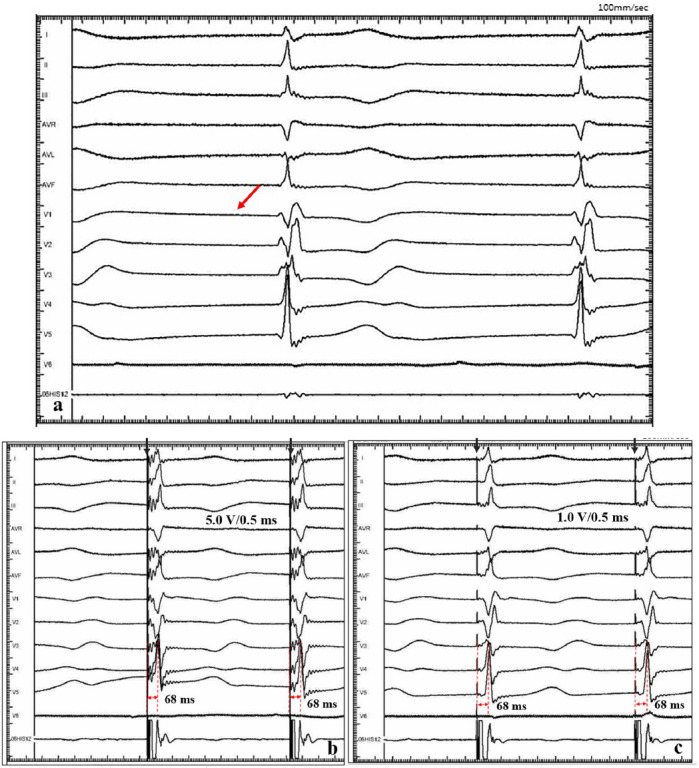
Intracardiac electrograms. **(a)** There was no atrial action potential (red arrow) in RA. **(b)** Ventricular pacing at 5.0 V/0.5 ms output, and Sti-LVAT was 68 ms. **(c)** Ventricular pacing at 1.0 V/0.5 ms output, and Sti-LVAT was 68 ms.

**FIGURE 4:**
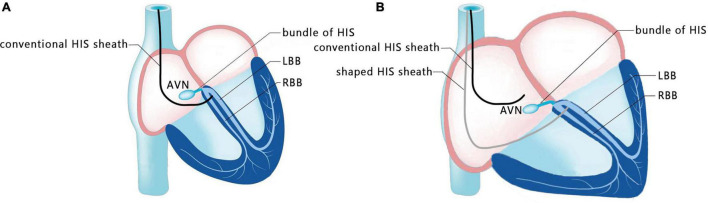
Schematic diagram of shaping the sheath. **(A)** Conventional HIS sheath in patients with a normal size atrium. **(B)** Shaped HIS sheath in patients with an enlarged atrium.

**FIGURE 5:**
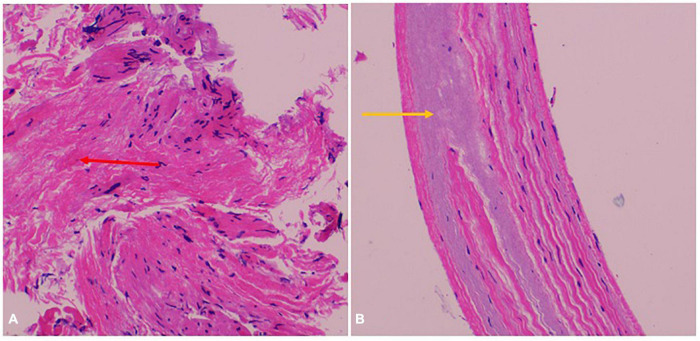
Myocardial biopsy pathological result. **(A)** Hyaline degeneration within the collagen fibers (red arrow). **(B)** Blurred stripes in some myocardial fibers with mucoid degeneration among parallel collagenous fibers (yellow arrow).

A 47-year-old female patient was admitted with recurrent syncope for 2 days. Bedside ECG on admission discovered no visible P waves or atrial fibrillatory waves, ventricular escape rhythm with ventricular rate 40–45 bpm, and torsade de pointes. Bedside echocardiography indicated an enlarged heart dominated by the atrium, where the right atrium (RA) size was 8.9 cm × 5.6 cm, the left atrium (LA) size was 7.2 cm × 5.6 cm, the left ventricular end diastolic diameter was 5.1 cm, the left ventricular ejection fraction (LVEF) was 50%, and there was extensive tricuspid regurgitation (Figure 1a). Moreover, only E waves were observed in the early diastolic period, but no A wave was detected in the late diastolic period in the mitral inflow pulse by Doppler recording (Figure 1b). A temporary pacemaker was immediately implanted. However, it was difficult for the temporary pacemaker lead to enter the right ventricle. The patient was instructed to take deep breaths and cough repeatedly. After repeated attempts, the lead was successfully placed into the apex of the right ventricle under the guidance of bedside echocardiography, and the pacing rate was set to 80 bpm. Subcutaneous injection of low molecular weight heparin was administered to prevent blood clots and intravenous infusion of cefazolin sodium was administered to prevent infection. The next day, ECG monitoring indicated ventricular pacing dysfunction, and the lead dislocation of the temporary pacemaker was considered. On the second day, a PPM was implanted. Intraoperative X-ray fluoroscopy showed that the temporary pacemaker lead was dislocated and coiled in the right atrial lumen (Figures 2a,b). Electrophysiology study indicated that no atrial action potential could be recorded in multiple regions of the RA, including right atrial appendage, middle atrial septum, the bottom of the interatrial septum, and low lateral region. Furthermore, there was a lack of atrial capture in several parts of the RA during high output at 5.0 V/0.5 ms, and consequently, atrial activity was considered to be paralyzed electrically (Figure 3a). The decisions to implant a single-chamber PPM and to attempt the LBBaP were made. First, a loach guide wire was delivered to the right ventricular outflow tract, and the His sheath (C315-His, Medtronic, Minneapolis, MN, United States) was delivered to the tricuspid annulus along the guide wire. The unshaped His sheath was difficult to be positioned in place. Through the sheath shaping technology, we put the inner core back into the His sheath, shaped the middle area of the second bend by hand, and adjusted the curvature of the His sheath. Subsequently, the sheath tube was sent to the tricuspid annulus, and an active fixation lead (3830, Medtronic, United States) was sent along the sheath to the right ventricular septum to select an acceptable initial fixation site with an obvious current of injury (Figures 4A,B). A right bundle branch block (RBBB) pattern was clearly observed during ventricular pacing when the lead was screwed into the interventricular septum, which indicated LBBaP (Figures 3b,c), with a stable stimulus to left ventricular activation time (Stim-LVAT) of 68 ms (a sensing of 11.8 mV, a pacing threshold of 0.9 V at a 0.5 ms pulse width and an impedance of 1,002 Ω). After the implantation of the pacemaker (ADSR01, Medtronic, Minneapolis, MN, United States), the temporary pacemaker leads were removed under fluoroscopy. The histology of myocardial biopsy showed hyaline degeneration within the collagen fibers, and the existence of blurred stripes in some myocardial fibers with mucoid degeneration among parallel collagenous fibers (Figure 5). A postoperative ECG showed that ventricular pacing was stable (VVI mode, pacing rate of 60 bpm). Benazepril 5 mg qd was given to improve cardiac remodeling, and apixaban 2.5 mg bid was given to prevent embolism. Ventricular tachycardia did not occur during the postoperative hospitalization. In the postoperative follow-up, the ventricular pacing burden was 96.2%. Pacemaker parameters remained stable with ventricular sensing of 12.5 mV and ventricular pacing threshold of 0.75 V at 0.4 ms. The patient has since remained free of syncope and not experienced cardiac insufficiency.

## Discussion

Atrial standstill is a rare condition defined by the lack of atrial electrical and mechanical activities, which may be intermittent or permanent, partial or total, and congenital or secondary. The congenital pathogenesis is mostly related to gene mutations including the reported mutations of *EMD*, *SCN5A*, and *MYL4* (6–9). The secondary causes are more commonly observed in patients with Emery-Dreifuss muscular dystrophy (10), cardiac sarcoidosis (11), acute myocarditis (12), acute myocardial infarction (13), hyperkalemia, drug poisoning (e.g., digoxin or quinidine), and surgical myocardial injury. The clinical manifestations of AS include dizziness, syncope (3), heart failure, arterial embolism, and stroke (14, 15). The mechanism of embolism is considered similar to that of atrial fibrillation. The loss of atrial regular contractile activity may lead to atrial thrombosis, which can cause arterial embolism. At present, there are no relevant guidelines and consensus to provide a treatment standard for AS. In this case, a long-term anticoagulant (apixaban) was given based on the benefits demonstrated by the relevant reports, and with the consent of the patient.

AS may manifest as partial or total atrial paralysis. It often appears early at the site of the high and mid-lateral RA, progresses to the entire RA, and then to the LA (16, 17). Bogossian et al. (18) reported a case of right atrial tachycardia despite silent RA with a remaining pacing site in the bottom of the interatrial septum. Demiralp et al. (19) reported a case of a partial AS with mechanical activity only documented at the left atrial appendage. For a few patients who had residual local electrical activity in the atrium, implanting atrial leads at appropriate sites can be used to select double-chamber pacemakers. Suzuki et al. (20) reported a case of AS with the atrial lead implanted in the coronary sinus. Considering the possibility of atrial disease progression, close follow-up is still recommended. Single-chamber PPM has been used in most previous cases. The torsade de pointes in this patient was considered to be secondary to QT interval prolongation in bradycardia, so we implanted a temporary pacemaker to increase the pacing rate to 80 bpm. After excluding other causes of torsade de pointes, a pacemaker was selected instead of an implantable cardiac defibrillator. We performed a single-chamber PPM implantation (VVI mode). During the postoperative hospitalization, ventricular tachycardia completely disappeared.

Cardiac pacing is the only effective treatment for symptomatic bradyarrhythmia. Traditional right ventricular apical pacing causes electrical and mechanical dyssynchrony resulting in left ventricular dysfunction, heart failure, and arrhythmias. Physiological pacing activates the normal cardiac conduction, thereby providing synchronized contractions of the ventricles. LBBaP technique is a novel pacing strategy evolving from His bundle pacing (HBP), including selective left bundle branch pacing (LBBP) and non-selective LBBP. The active lead is twisted through the septum from the right ventricular septum to the left fascicular branch area under the intima of the left ventricular septum, and the pacing captures the left Purkinje network to circumvent the blocked site and maintains the electrical synchronization of the left ventricle (21–24). LBBaP paces beyond the site of block and results in a low pacing threshold with a high success rate in patients with infranodal atrioventricular block. Relevant studies have reported a success rate of 81–93% (24–26). Huang et al. (27) reported the first case of LBBaP. The left bundle branch block (LBBB) could not be corrected at 10 V/0.5 ms output by HBP. The tip end of the electrode wire was then sent to the distal end of the ventricle, and as a result, the LBBB could be corrected by 0.5 V/0.5 ms output. The pacing threshold was low and stable, and above all, heart failure symptoms improved significantly during follow-up. Since then, the characteristics of LBBaP have been continuously explored. An *in vivo* canine model illustrated the electrophysiological parameters and anatomical evaluation of LBBP, and showed the improvement of hemodynamics (28). LBBaP was confirmed to maintain left ventricular synchronization by nuclide examination (29). Several clinical studies have also confirmed the benefits of LBBaP. Notably, a multicenter observational study verified that LBBaP improved cardiac function and reduced the hospitalization rate of heart failure patients. It also showed better clinical outcomes than right ventricular pacing (RVP) in patients with atrioventricular block, requiring a heavy burden of ventricular pacing (30). For patients with heart failure, current studies showed that LBBaP was associated with remarkable improvements in cardiac function, mechanical synchronization, and mechanical efficiency and may be a promising alternative to cardiac resynchronization therapy (31–35). In addition, many clinical studies have demonstrated the safety of LBBaP. A single-center study indicated that the total incidence of procedure-related complications of LBBP was 1.63% (36). Another single-center study showed that the complications and cardiac outcomes were not significantly different between LBBP and RVP after mid-long-term follow-up (37).

Huang et al. (21) expounded the operation specification of LBBP for the first time, and more admissible judgment criteria were provided in the subsequent studies. Su et al. (38) indicated that the current of injury is meanful to judge the LBB capture, pacing threshold and electrode perforation. Huang et al. (39) established the standard model for judging the capture of LBB. Direct LBB capture was defined as retrograde HIS potential on the HPV lead and/or anterograde left conduction system potentials on the multielectrode catheter during LBBP. An abrupt decrease in Stim-LVAT of ≥10 ms and demonstration of selective LBBP could be used as simple criteria to confirm LBB capture. In this present case, we recognized this as an LBBaP by the following evidences: ① paced morphology is a RBBB shape; ② the Sti-LVAT remains 68 ms at high and low outputs; ③ paced morphology was not a typical RBBB shape and the dissociation was not definite enough at 1.0 V/0.5 ms output. Because of AS and ventricular escape rhythm without normal AV conduction in this case, the intracardiac electrograms did not show the internal rhythm and the LBB potential. Retrograde His potential or anterograde left conduction system potentials are a golden criterion of direct LBB captured, but it is not practical and adaptable in clinical practice, especially in complex conditions like in our case. Zhang et al. (40) explored a simplified approach (“9-partition method”) to perform LBBaP under fluoroscopy. This provides a means of operation in the absence of multi-channel electrophysiology instruments. For special anatomical structures, techniques such as the “sheath in sheath” (His bundle sheath covering the left ventricular delivery system) (25, 34, 41) can also be used to further increase the supporting force of the sheath and to contribute to the fixation of leads in the target area. In this case, we shaped the middle area of the second bend of the His sheath by hand, and adjusted the curvature to reach the acceptable initial site. In recent years, the use of newly developed implant tools and related auxiliary means have helped clinicians to implant pacemaker systems into patients with special anatomical structures.

In summary, when PPM treatment is essential in AS with a giant atrium, LBBaP is a promising pacing strategy and the technique should not be waived prematurely. In this case, LBBaP was successfully achieved by HIS sheath shaping, and the intraoperative and postoperative parameters were satisfactory and stable. Further follow-up observation is indispensable to assess the stability, safety, and clinical prognosis. This clinical experience in such exceptional circumstances justifies further investigation.

## Data Availability Statement

The original contributions presented in the study are included in the article/supplementary material, further inquiries can be directed to the corresponding author.

## Ethics Statement

Written informed consent was obtained from the individual(s) for the publication of any potentially identifiable images or data included in this article.

## Author Contributions

JinZ and QJ contributed to the conception of the study. JinZ wrote the manuscript. QY, JiaZ, and QC helped to analyze the patient data and curate the data. QJ helped to perform the analysis with constructive discussions. All authors contributed to the article and approved the submitted version.

## Conflict of Interest

The authors declare that the research was conducted in the absence of any commercial or financial relationships that could be construed as a potential conflict of interest.

## Publisher’s Note

All claims expressed in this article are solely those of the authors and do not necessarily represent those of their affiliated organizations, or those of the publisher, the editors and the reviewers. Any product that may be evaluated in this article, or claim that may be made by its manufacturer, is not guaranteed or endorsed by the publisher.

## References

[1] Parascandola J. Arthur Cushny, optical isomerism, and the mechanism of drug action. *J Hist Biol.* (1975) 8:145–65. 10.1007/BF00130436 11609891

[2] Harley A. Persistent right atrial standstill. *Br Heart J.* (1976) 38:646–9. 10.1136/hrt.38.6.646 132178 PMC483049

[3] Ahmad Y Akbar S Mohammad S Ali M. Atrial standstill: a rare case. *J Tehran Heart Cent.* (2011) 6:152–4. 23074623 PMC3466890

[4] Amir FF Arash A Majid H Mohammad AS. Familial atrial standstill in association with dilated cardiomyopathy. *Pacing Clin Electrophysiol.* (2005) 28:1005–8. 10.1111/j.1540-8159.2005.00198.x 16176547

[5] Lan S Songjie W Shengjie W Lei X Zhouqing H Xiao C Long-term safety and feasibility of left bundle branch pacing in a large single-center study. *Circ Arrhythm Electrophysiol.* (2021) 14:e009261. 10.1161/CIRCEP.120.009261 33426907

[6] Giuseppe B Elena B Matteo Z Vincenzo LM Marco V Marisa T Cardiolaminopathies from bench to bedside: challenges in clinical decision-making with focus on arrhythmia-related outcomes. *Nucleus.* (2018) 9:442–59. 10.1080/19491034.2018.1506680 30130999 PMC6244733

[7] Kumar S Baldinger SH Gandjbakhch E Maury P Sellal JM Alexander FA Longterm arrhythmic and nonarrhythmic outcomes of lamin A/C mutation carriers. *J Am Coll Cardiol.* (2016) 68:2299–307. 10.1016/j.jacc.2016.08.058 27884249

[8] Gollob MH. Expanding the clinical phenotype of emerinopathies: atrial standstill and left ventricular noncompaction. *Circ Arrhythm Electrophysiol.* (2020) 13:e009338. 10.1161/CIRCEP.120.009338 33079577

[9] Makita N Sasaki K Groenewegen WA Yokota T Yokoshiki H Murakami T Congenital atrial standstill associated with coinheritance of a novel SCN5A mutation and connexin 40 polymorphisms. *Heart Rhythm.* (2005) 2:1128–34. 10.1161/CIRCEP.120.009338 16188595

[10] Boriani G Gallina M Merlini L Bonne G Toniolo D Amatie S Clinical relevance of atrial fibrillation/flutter, stroke, pacemaker implant, and heart failure in Emery-Dreifuss muscular dystrophy: a long-term longitudinal study. *Stroke.* (2003) 34:901–8. 10.1161/01.STR.0000064322.47667.49 12649505

[11] Kim TH Kim H Park HS Han S Park NH. Atrial standstill in suspected isolated cardiac sarcoidosis. *J Cardiol Cases.* (2016) 14:136–8. 10.1016/j.jccase.2016.06.010 30546677 PMC6283722

[12] Prabhu MA Srinivas Prasad BV Thajudeen A Namboodiri N. Persistent atrial standstill in acute myocarditis. *Indian Pediatr.* (2016) 53:162–4. 10.1007/s13312-016-0814-3 26897154

[13] Koshimizu TA Komori S Ishihara T Kohno I Umetani K Sawanobori T Restored atrial excitability after late recanalization in a patient with atrial standstill and acute myocardial infarction. *Pacing Clin Electrophysiol.* (2002) 25:217–9. 10.1046/j.1460-9592.2002.00217.x 11915991

[14] Agnethe MA Victoria EK Jesper IR Malene LB. Atrial standstill presenting as cerebral infarction in a 7-year-old girl. *SAGE Open Med Case Rep.* (2019) 4:2050313. 10.1177/2050313X19827735 30783526 PMC6366293

[15] Taisuke I Hiroyuki M Julien B Masanori PT Keiichi H Shigenori T Cardiac emerinopathy: a nonsyndromic nuclear envelopathy with increased risk of thromboembolic stroke due to progressive atrial standstill and left ventricular noncompaction. *Circ Arrhythm Electrophysiol.* (2020) 13:e008712. 10.1161/CIRCEP.120.008712 32755394

[16] Nakazato Y Nakata Y Hisaoka T Sumiyoshi M Ogura S Yamaguchi H. Clinical and electrophysiological characteristics of atrial standstill. *Pacing Clin Electrophysiol.* (1995) 18:1244–54. 10.1111/j.1540-8159.1995.tb06964.x 7659578

[17] Lévy S Pouget B Bemurat M Lacaze JC Clementy J Bricaud H Partial atrial electrical standstill: report of three cases and review of clinical and electrophysiological features. *Eur Heart J.* (1980) 1:107–16. 10.1093/oxfordjournals.eurheartj.a061104 7285968

[18] Bogossian H Frommeyer G Lemke B Zarse M. Right atrial tachycardia despite silent right atrium: a case report and review of the literature. *Clin Res Cardiol.* (2015) 104:185–8. 10.1007/s00392-014-0761-8 25213706

[19] Demiralp E Kirilmaz A Cebeci BS Ulusoy RE. Partial atrial standstill: a case report. *J Electrocardiol.* (2005) 38:252–5. 10.1016/j.jelectrocard.2005.01.009 16003711

[20] Suzuki Y Takei A Takahara H Taniguchi Y Ozawa T Inoue N. A case of atrial standstill with the atrial lead of a dual-chamber pacemaker implanted in the coronary sinus. *HeartRhythm Case Rep.* (2019) 5:338–42. 10.1016/j.hrcr.2019.03.008 31285994 PMC6587059

[21] Weijian H Xueying C Lan S Shengjie W Xue X Pugazhendhi V. A beginner‘s guide to permanent left bundle branch pacing. *Heart Rhythm.* (2019) 16:1791–6. 10.1016/j.hrthm.2019.06.016 31233818

[22] Peng L Qiaozhu W Hongke S Xinghua Q Qiangsun Z. Left bundle branch pacing: current knowledge and future prospects. *Front Cardiovasc Med.* (2021) 23:630399. 10.3389/fcvm.2021.630399 33834042 PMC8021709

[23] Shu Z Xiaohong Z Michael RG. Left bundle branch pacing: JACC review topic of the week. *J Am Coll Cardiol.* (2019) 74:3039–49. 10.1016/j.jacc.2019.10.039 31865972

[24] Li Y Chen K Dai Y Li C Sun Q Chen R Left bundle branch pacing for symptomatic bradycardia: implant success rate, safety, and pacing characteristics. *Heart Rhythm.* (2019) 16:1758–65. 10.1016/j.hrthm.2019.05.014 31125667

[25] Vijayaraman P Subzposh FA Naperkowski A Panikkath R John K Mascarenhas V Prospective evaluation of feasibility and electrophysiologic and echocardiographic characteristics of left bundle branch area pacing. *Heart Rhythm.* (2019) 16:1774–82. 10.1016/j.hrthm.2019.05.011 31136869

[26] Hua W Fan X Li X Niu H Gu M Ning X Comparison of left bundle branch and his bundle pacing in bradycardia patients. *JACC Clin Electrophysiol.* (2020) 6:1291–9. 10.1016/j.jacep.2020.05.008 33092757

[27] Huang W Su L Wu S Lei X Xiao F Zhou X . A novel pacing strategy with low and stable output: pacing the left bundle branch immediately beyond the conduction block. *Can J Cardiol.* (2017) 33:1736.e1–3. 10.1016/j.cjca.2017.09.013 29173611

[28] Chen X Jin Q Li B Jia J Sharma PS Huang W Electrophysiological parameters and anatomical evaluation of left bundle branch pacing in an in vivo canine model. *J Cardiovasc Electrophysiol.* (2020) 31:214–9. 10.1111/jce.14300 31778271

[29] Hou X Qian Z Wang Y Qiu Y Chen X Jiang H Feasibility and cardiac synchrony of permanent left bundle branch pacing through the interventricular septum. *Europace.* (2019) 21:1694–702. 10.1093/europace/euz188 31322651

[30] Li X Zhang J Qiu C Wang Z Li H Pang K Clinical outcomes in patients with left bundle branch area pacing vs. right ventricular pacing for atrioventricular block. *Front Cardiovasc Med.* (2021) 8:685253. 10.3389/fcvm.2021.685253 34307499 PMC8297826

[31] Wu S Su L Vijayaraman P Zheng R Cai M Xu L Left bundle branch pacing for cardiac resynchronization therapy: nonrandomized on-treatment comparison with his bundle pacing and biventricular pacing. *Can J Cardiol.* (2021) 37:319–28. 10.1016/j.cjca.2020.04.037 32387225

[32] Liu W Hu C Wang Y Cheng Y Zhao Y Liu Y Mechanical synchrony and myocardial work in heart failure patients with left bundle branch area pacing and comparison with biventricular pacing. *Front Cardiovasc Med.* (2021) 20:727611. 10.3389/fcvm.2021.727611 34490382 PMC8417592

[33] Liu J Sun F Wang Z Sun J Jiang X Zhao W Left bundle branch area pacing vs. biventricular pacing for cardiac resynchronization therapy: a meta-analysis. *Front Cardiovasc Med.* (2021) 24:669301. 10.3389/fcvm.2021.669301 34109227 PMC8180564

[34] Huang W Wu S Vijayaraman P Su L Chen X Cai B Cardiac resynchronization therapy in patients with nonischemic cardiomyopathy using left bundle branch pacing. *JACC Clin Electrophysiol.* (2020) 6:849–58. 10.1016/j.jacep.2020.04.011 32703568

[35] Chen X Jin Q Bai J Wang W Qin S Wang J The feasibility and safety of left bundle branch pacing vs. right ventricular pacing after mid-long-term follow-up: a single-centre experience. *Europace.* (2020) 22(Suppl. 2):ii36–44. 10.1093/europace/euaa294 33370799

[36] Ye Y Wu S Su L Sheng X Zhang J Wang B Feasibility and outcomes of upgrading to left bundle branch pacing in patients with pacing-induced cardiomyopathy and infranodal atrioventricular block. *Front Cardiovasc Med.* (2021) 8:674452. 10.3389/fcvm.2021.674452 34195236 PMC8236829

[37] Chen X Wei L Bai J Wang W Qin S Wang J Procedure-related complications of left bundle branch pacing: a single-center experience. *Front Cardiovasc Med.* (2021) 8:645947. 10.3389/fcvm.2021.645947 33869306 PMC8044788

[38] Su L Xu T Cai M Xu L Vijayaraman P Sharma PS Electrophysiological characteristics and clinical values of left bundle branch current of injury in left bundle branch pacing. *J Cardiovasc Electrophysiol.* (2020) 31:834–42. 10.1111/jce.14377 32009260

[39] Wu S Chen X Wang S Xu L Xiao F Huang Z Evaluation of the criteria to distinguish left bundle branch pacing from left ventricular septal pacing. *JACC Clin Electrophysiol.* (2021) 7:1166–77. 10.1016/j.jacep.2021.02.018 33933414

[40] Zhang J Wang Z Zu L Cheng L Su R Wang X Simplifying physiological left bundle branch area pacing using a new nine-partition method. *Can J Cardiol.* (2021) 37:329–38. 10.1016/j.cjca.2020.05.011 32428620

[41] Huang W Zhou X Ellenbogen KA. Pursue physiological pacing therapy –a better understanding of left bundle branch pacing and left ventricular septal myocardial pacing. *Heart Rhythm.* (2021) 18:1290–1. 10.1016/j.hrthm.2021.05.013 33992731

